# Pathways From Social Activities to Cognitive Functioning: The Role of Physical Activity and Mental Health

**DOI:** 10.1093/geroni/igaa015

**Published:** 2020-06-30

**Authors:** Ella Cohn-Schwartz

**Affiliations:** Department of Public Health, Faculty of Health Sciences, Ben-Gurion University of the Negev, Be’er Sheva, Israel

**Keywords:** Cognition, Depression, Exercise/Physical Activity, Social activity

## Abstract

**Background and Objectives:**

One of the greatest challenges of old age is the risk of cognitive decline. Engagement in social activities has been identified as a possible protective factor. However, it is not yet clear what are the mechanisms underlying this association. This study aims to elucidate the pathways through which social activities impact cognitive functioning, focusing on physical activity and mental health as possible mediators.

**Research Design and Methods:**

The study utilized 3 waves of data—the fourth, fifth, and sixth waves of the Survey of Health, Ageing and Retirement in Europe, collected in 2011, 2013, and 2015, respectively. It focused on respondents aged 60 and older. Cognitive functioning was assessed via immediate recall, delayed recall, and fluency. Social activities were measured by volunteering and attending social clubs. Data were analyzed using a structural equation modeling approach.

**Results:**

The results indicated a significant direct effect of social activities on cognitive functioning. That is, being socially active at baseline was related to better cognitive function 4 years later. The results also indicated the existence of indirect effects. Engaging in social activities was related to better mental health and more physical activities 2 years later, which were related to better subsequent cognitive performance.

**Discussion and Implications:**

These findings highlight the mediating roles of physical activity and mental health in the effects of social activities on cognitive functioning. Understanding these mechanisms can help optimize social activity interventions to improve cognitive aging.

**Translational Significance:** The findings stress that social activities are associated with increases in physical activity and improvements in mental health, both of which contribute to better cognitive function. These results can encourage practitioners to promote participation in social activities to improve the cognitive function of older adults. These findings can also guide practitioners to pay particular attention to the mental health and physical health aspects of such social activities.

One of the greatest challenges of old age is the risk of cognitive decline, with poor cognitive functioning having far-reaching implications for everyday life. Cognitive decline is a common part of aging (Deary et al., 2009) and is associated with reduced functional capacity, loss of independence (Luppa et al., 2010), and mortality (Schupf et al., 2005). Therefore, finding effective population-based strategies to improve cognitive performance in old age is an increasingly important public health priority. The cognitive enrichment hypothesis supports the potentially beneficial effects of such strategies by claiming that although cognitive losses tend to become more pronounced with advancing age, cognitive functioning can still be improved by individuals’ behaviors and lifestyles (Hertzog, Kramer, Wilson, & Lindenberger, 2009). The hypothesis claims that the brains of older adults can adapt to compensate for neural changes in old age (Park & Reuter-Lorenz, 2009) and that this adaptation can be influenced by individuals’ social context and personal behavior. This perspective provides an overarching framework regarding the importance of assessing social factors that can improve cognitive function in old age.

Engagement in social leisure activities has been previously identified as a factor that can help older adults maintain their cognitive status (Barnes, Mendes De Leon, Wilson, Bienias, & Evans, 2004; Bourassa, Memel, Woolverton, & Sbarra, 2017; Haslam, Cruwys, & Haslam, 2014; Hwang, Park, & Kim, 2018; D. Kim, Arai, & Kim, 2017). However, it is not yet clear what are the mechanisms underlying this association. The cascading causal process model of social integration and health suggests several potential pathways between a socially active life and health (Berkman, Glass, Brissette, & Seeman, 2000). Guided by this model, the current study sets out to elucidate the pathways through which social activities can impact cognitive functioning among older adults, focusing on physical activity and mental health.

Research has generally identified social activities as a protective factor against cognitive decline, and several longitudinal studies indicated that engagement in social activities is associated with less cognitive decline over time (Barnes et al., 2004; Bourassa et al., 2017; Haslam et al., 2014; Hwang et al., 2018; James, Wilson, Barnes, & Bennett, 2011; D. Kim et al., 2017), although this has not always been found (Marioni et al., 2015). For example, participating in a higher number of social activities, including activities or groups outside the home, was associated with a reduced rate of cognitive decline. A high (90th percentile) level of high social engagement reduced the rate of decline by 91% compared with a low level (10th percentile; Barnes et al., 2004). Participation in group activities was also related to better subsequent cognitive function (Haslam et al., 2014). Additionally, engagement in social activities, assessed as composite measures of the variety and frequency of engagement in activities, such as attending social events and volunteering, was related to a lower risk of progression from mild to severe cognitive impairment among mildly impaired adults (Hughes, Flatt, Fu, Chang, & Ganguli, 2013).

However, it is not yet clear how social activities affect cognition in old age, despite the importance of this topic. The cascading causal process model of social integration and health, described by Berkman and colleagues (2000), offers a theoretical framework that explains these effects. The model maintains that leading a socially active life can affect one’s health through various pathways. One pathway is the health-behavioral pathway, suggesting that social activities are related to health-promoting behaviors, including physical exercise. Social activities can induce a more physically active life by ambulation to and from the activity, participation in trips or sports and providing companions for physical activities. The psychological pathway suggests an impact through reducing depression and increasing well-being (Berkman et al., 2000). These activities can provide enjoyment, improve one’s mood, present a context for older adults to share rewarding moments with others and allow adults to invest time and energy in their interests. A similar focus on the psychological pathway is presented by Flatt and Hughes (2013). In their perspective article, they present a conceptual model which suggests that social activities are important for cognitive health by leading to lower depression. However, while these models offer a promising framework to understand the impact of social activities, less attention has been paid to empirically explore the mediating mechanisms underlying the enrichment effects of social involvement (Hertzog et al., 2009). Therefore, the current study will examine two possible mechanisms—physical activity and mental health.

The first mechanism to be explored is physical activity. Empirical evidence provides some support for the premise that social activities promote physical exercise. Volunteering, for example, was shown to increase physical activity in urban older adults who were previously inactive, perhaps by traveling to and from the activity and by having the volunteering itself include walking and being physically active (Tan, Xue, Li, Carlson, & Fried, 2006). Accordingly, the World Health Organization (WHO) cites sports or social clubs as favorable environments to promote physical activity (WHO, 1996). Engaging in physical exercise, in turn, is considered to be part of an enriching lifestyle that can facilitate cognitive functioning (Hertzog et al., 2009). Numerous studies have shown physical activity to be associated with a decreased risk of cognitive decline (Baumgart et al., 2015; Blondell, Hammersley-Mather, & Veerman, 2014; Colcombe & Kramer, 2004; Sofi et al., 2011). In their meta-analysis, Sofi and colleagues (2011) demonstrated that individuals who engaged in low-to-moderate levels of physical activity at baseline had a 35% reduced risk of developing cognitive decline at follow-up, compared with those who did not engage in physical activities.

Social activities can also operate through a psychological avenue, by being enjoyable and meaningful experiences that improve individuals’ mental health, consequently leading to better cognition. Previous research showed that social activities such as volunteering and going to sport events are associated with lower depression trajectories (Hong, Hasche, & Bowland, 2009). Psychological factors can subsequently affect cognitive performance. Cognition might be hampered by negative affect, while positive attitudes and beliefs can be positively linked to late-life cognition (Hertzog et al., 2009). Accordingly, higher levels of depressive symptoms were found to be associated with more rapid cognitive decline among older adults (van den Kommer et al., 2013; Wilson et al., 2014).

Social activities in themselves can also directly impact one’s cognitive functioning, independently of their physical and emotional aspects. The “use it or lose it” theory offers another, more direct explanation for the effects of social activities. This theory suggests that frequent engagement with others provides “cognitive exercise,” which stimulates the mind and preserves cognitive functions (Hultsch, Hertzog, Small, & Dixon, 1999). Engaging in social activities can be cognitively stimulating, possibly accounting for some of its protective effect against cognitive decline (Schwartz, Khalaila, & Litwin, 2019). Cognitive stimulation may be achieved by organizing social gatherings, navigating social cues, and engaging in complex conversations (Bielak, 2010; E. Y. Kim & Kim, 2014). Such encounters can also enhance cognitive reserve, which allows people to tolerate brain pathology without showing clear behavioral symptoms (Bennett, Schneider, Tang, Arnold, & Wilson, 2006). Therefore, the present study will explore the direct pathway between social activities and cognitive functioning, alongside indirect pathways through mental health and physical activity.

To sum, the current study provides an in-depth exploration of the linkage between social activities and cognitive functioning. To better understand this association, it considers both the direct and the indirect pathways by examining whether initial levels of social activities are related to subsequent cognitive functioning via increased physical activity and improved mental health. The study will utilize three data points, allowing for a longitudinal examination of the mediation mechanisms (Cole & Maxwell, 2003). It will address three hypotheses.

1. Social activities are positively associated with subsequent cognitive functioning.
2. Social activities are also indirectly linked with cognitive functioning. That is, social activities are positively associated with future physical activity, which is related to higher cognitive functioning among older adults.
3. Social activities are also associated with future mental health, which is related to higher cognitive functioning among older adults.

## Research Design and Methods

### Participants and Procedure

Data were taken from the Survey of Health, Ageing and Retirement in Europe (SHARE), a panel population-based representative survey focusing on community-dwelling European adults aged 50 and older and their spouses of any age. Data have been collected biennially, beginning in 2004, using a computer-assisted personal interviewing program, supplemented by a self-completed paper-and-pencil questionnaire (Börsch-Supan et al., 2013). The current study considered respondents aged 60 and older. It utilized three waves of data—the fourth, fifth, and sixth waves collected in 2011, 2013, and 2015, respectively. In the fourth wave of SHARE (2011), the sample size was substantially increased by more than 20,000 respondents due to the inclusion of four new countries and drawing refreshment samples in most of the established countries (Börsch-Supan et al., 2013). Using Waves 4–6 allowed the study to be based on a larger sample and enabled a longitudinal mediation analysis. The current study included data from 12 European countries (Austria, Belgium, Czech Republic, Denmark, Estonia, France, Germany, Italy, Slovenia, Spain, Sweden, and Switzerland). The study sample included participants who took part in all three waves, resulting in a sample size of 19,997 respondents. Table 1 presents the descriptive characteristics of the sample. The average age in the sample at baseline was 70, and 57% were women. Over half of the sample had secondary education or above, and their health and hearing were rated as “Good” (3 out of 5).

**Table 1.** Sample Characteristics of the Study

Variable	*M* (*SD*)	%	Range
Age	70.11 (7.29)		60–101
Gender (women)		57.1	
Education (secondary and above)		56.4	
Self-perceived health	2.76 (1.04)		1–5
Hearing	2.76 (1.03)		1–5
Financial status	3.00 (0.96)		1–4
Sports or social club T1		17.4	
Volunteering T1		28.1	
Moderate physical activity T1	3.42 (1.02)		1–4
Moderate physical activity T2	3.32 (1.09)		1–4
Vigorous physical activity T1	2.28 (1.32)		1–4
Vigorous physical activity T2	2.21 (1.31)		1–4
Depressive symptoms T1	2.44 (2.18)		0–12
Depressive symptoms T2	2.45 (2.22)		0–12
Quality of life T1	37.41 (6.27)		15–48
Quality of life T2	37.51 (6.30)		12–48
Immediate recall T1	5.08 (1.78)		0–10
Immediate recall T3	5.04 (1.81)		0–10
Delayed recall T1	3.64 (2.13)		0–10
Delayed recall T3	3.65 (2.21)		0–10
Fluency T1	19.76 (7.24)		0–45
Fluency T3	19.67 (7.39)		0–45

### Measures

#### Independent variable

Social activities were measured using two questions. The first question probed if the respondent had done voluntary or charity work in the 12 months prior to the interview, and the second question inquired about participation in a sport, social or other kinds of club. Thus, they were coded as dummy variables indicating the participation or lack of participation in such activities. The SHARE data set contains a question about an additional social activity—participation in political organizations. However, preliminary analyses showed that this additional variable had a lower loading on the latent factor of social activities (<0.4), and it was therefore not used in the study model.

#### Dependent variables

Cognitive functioning was assessed via three cognitive tests that are sensitive to aging-related decline (Dewey & Prince, 2005). Immediate recall was measured using a modified version of the Rey’s Auditory Verbal Learning Test (Dal Bianco, Garrouste, & Paccagnella, 2013). The test examines how many of 10 words the respondent was able to recall immediately after the interviewer read the words. The task has a score range of 0–10. Delayed recall measured the respondent’s ability to recall the same words later on, after other interview questions. This measure also has a score range of 0–10. The immediate and delayed recall tasks test short-term verbal learning and memory as well as information retention (Dal Bianco et al., 2013) and can be used as a measure of episodic memory (Cheke & Clayton, 2013). Fluency measures how many distinct animals the respondent can name, without repetitions or proper nouns, in a 1-min period. It is considered a measure of executive functioning, but also includes other processes, such as semantic memory and processing speed (Clark et al., 2009; Haugrud, Crossley, & Vrbancic 2011). Due to outliers in a small number of cases, scores greater than 45 were recoded as 45. Six respondents whose scores fell more than 3 standard deviations above the mean group score (i.e., greater than 45) were given a score of 45.

These measures were used to assess cognitive function, as it is recognized that free recall tasks and the fluency task are sensitive to cognitive aging (Souchay, Isingrini & Espagnet, 2000). Principal component factor analysis using varimax rotation was performed, showing that these three variables load on a single factor, explaining 70% of the variance at T1 and 74% of the variance at T3. The results allowed for the use of these three variables as the components of a latent factor in the main analyses.

#### Mediators

Mental health was captured by means of depressive symptoms and quality of life, representing the negative and positive aspects of mental health. Depressive symptoms were assessed using the EURO-D scale, developed in order to measure late-life depressive symptoms across European countries (Prince et al., 1999). It consists of 12 binary items inquiring about different depressive symptoms, such as depressed mood, pessimism, and irritability. Scores sum these symptoms and range from 0 to 12, with higher scores indicating greater symptoms severity. The scale was shown to have good validity and internal consistency (Castro-Costa et al., 2008). In the current study, internal reliability, measured by Kuder-Richardson’s *p*, was .77 at T1 and .70 at T2. Quality of life was assessed using the CASP-12 scale (Knesebeck, Wahrendorf, Hyde, & Siegrist, 2007). The scale is made up of 12 items in total, divided into four subscales measuring control, autonomy, self-realization, and pleasure. Each subscale contains three items ranging from 1 (*often*) to 4 (*never*). Several items are phrased in a manner which reflects poor quality of life (e.g., “How often do you feel left out of things?”). These items were reverse-coded so that a higher score on all items meant the higher quality of life (total range of the scale = 12–48). Cronbach’s alpha reliability coefficient in this study was .80 at T1 and .81 at T2.

The second mediator was physical activity. This was measured using the two variables available in the SHARE data which inquire about the frequency of engaging in moderate and vigorous physical activities: “How often do you engage in activities that require a moderate level of energy such as gardening, cleaning the car, or doing a walk?” and “How often do you engage in vigorous physical activity, such as sports, heavy housework, or a job that involves physical labour?” Response options ranged from 1 (*more than a week*) to 4 (*hardly ever, or never*) and were recoded such that a higher score indicates a greater frequency of engaging in physical activity.

#### Covariates

Several sociodemographic and health covariates were controlled for in the analyses due to their possible associations with cognitive functioning. Age was used as a continuous variable. Gender was coded as 0 (*men*) and 1 (*women*). The level of education was based on the standardized coding of the International Standard Classification of Education (ISCED-97). It was measured in this analysis as (0) partial secondary schooling or less (ISCED-97 = 0–2); and (1) secondary schooling or more (ISCED-97 = 3–6). Health was measured by asking respondents to rate their health, on a Likert scale of 1–5. The scale was recoded such that higher scores indicate better self-rated health.

### Statistical Analyses

Data were analyzed using a structural equation modeling (SEM) approach, with the package Lavaan in R (Rosseel, 2012). The study variables of cognitive functioning, social activities, mental health, and physical activity were modeled as latent variables to account for measurement error. For each latent factor, the first path loading was set to 1. The longitudinal design of this study permitted assessing the predictor and mediating factors before the outcome variable of T3 cognitive function while controlling for previous levels of cognition, thus reducing the risk of bias in estimating predictor–outcome associations at the same time point. Significance of the mediation hypotheses was evaluated using the Delta method (Sobel, 1982). This analysis uses a test of the product of the coefficients relating the independent variable to the mediator (α), and the mediator to the dependent variable (β). This approach tests the significance of the indirect effect by dividing the estimate of the indirect effect, the product αβ by its standard error and comparing this value to a standard normal distribution (Sobel, 1982). Factorial invariance over time was achieved by setting factor loadings and intercepts to equality at the two measurements for the latent factors. Full information maximum likelihood was used to handle missing data. The amount of missingness was relatively low. The models were run with a maximum likelihood estimator with robust standard errors to allow variables to deviate from multivariate normality (Satorra & Bentler, 2010). Model fit was evaluated primarily based on the criteria of comparative fit index (CFI) > .95, standardized root mean square residual (SRMR) < .08, and root mean square error of approximation (RMSEA) < .08 (Hooper, Coughlan, & Mullen, 2008).

The analysis began with a measurement model that explored the covariances between the latent factors in the study. Following the establishment of a measurement model, a series of structural models was estimated by adding predictive paths between the study variables. These models examined each effect separately, before examining them in a single final model. The first model tested only the relationship between the T1 social activities and T3 cognitive function. The second model added physical activity at T2 as a mediator between T1 social activities and T3 cognitive function. The third model included mental health at T2 as a mediator between T1 social activities and T3 cognitive function. The final model included both mediators and the direct association to test for mediation effects (Zhao, Lynch & Chen, 2010). In the final model, cognitive function at T3 was predicted using social activities at baseline and mental health and physical activity at T2. Mental health and physical activity at T2 were predicted using cognitive function, social activities, mental health, and physical activity at baseline. The study variables were also regressed on the baseline covariates. The model included covariances between the variables at T1 and covariance between the mediators at T2. Error terms were allowed to covary among matching factor loadings at the two time points.

## Results

Table 2 presents correlations among the observed variables that indicate social activities, cognitive function, physical activities, and mental health. All the variables are shown at baseline for clarity. For each latent construct, the observed indicators were all intercorrelated (e.g., depressive symptoms were correlated with quality of life), supporting their use as factors in the latent constructs. Additionally, the observed social activity variables were correlated with the variables indicating physical activity and mental health, as well as with the cognitive variables, in accordance with the study’s hypotheses. The mediating variables were also significantly linked to cognitive performance.

**Table 2.** Correlations Among Observed Variables at Baseline (T1)

	Volunteering	Moderate physical activity	Vigorous physical activity	Depressive symptoms	Quality of life	Immediate recall	Delayed recall	Fluency
Sports or social club	.22***	.11***	.11***	−.10***	.19***	.15***	.16***	.14***
Volunteering	—	.16***	.21***	−.14***	.23***	.17***	.18***	.19***
Moderate physical activity		—	.38***	−.23***	.28***	.20***	.17***	.23***
Vigorous physical activity			—	−.20***	.25***	.19***	.18***	.23***
Depressive symptoms				—	−.53***	−.18***	−.19***	−.20***
Quality of life					—	.25***	.26***	.26***
Immediate recall						—	.71***	.48***
Delayed recall							—	.44***

***p < .001.

**Figure 1.**
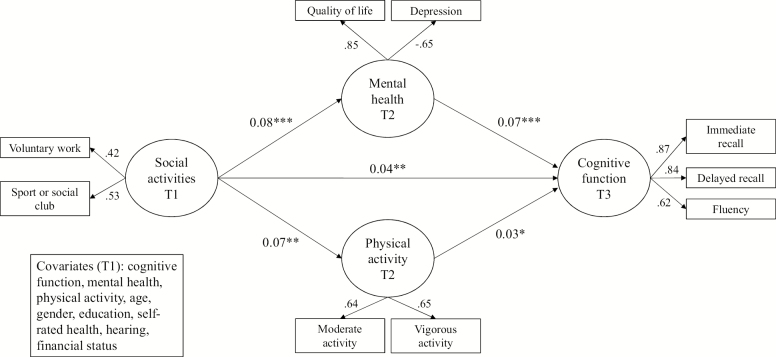
Structural equation modeling model of mental health and physical activity as mediators in the relationship between social activities and cognitive functioning. *Notes.* The following paths were omitted in the figure to increase figure legibility: baseline covariates, covariances between the variables at T1, covariances between the mediators at T2; paths present standardized coefficients; * *p* < .05, ** *p* < .01, *** *p* < .001.

In order to estimate the SEM model of interest, the analysis began with a measurement model. This model had good fit to the data [χ ^2^ (76) = 2,492.45, CFI = .98, RMSEA = .040 (confidence interval [CI] = 0.039–0.041), SRMR = .04]. A series of structural models followed the establishment of a measurement model. The first model showed that social activities had a significant association with cognitive function at T3 (*p* < .001). The second model added physical activities as a mediator and found a significant mediation pathway [*B* (*SE*) = 0.04 (0.01), *p* = .003]. The third model added the mediation of mental health (without the mediation of physical activities) and indicated that this pathway was also significant [*B* (*SE*) = 0.07 (0.01), < .001]. The final model incorporated the direct and indirect pathways of interest (Figure 1 and Table 3). That model showed good fit to the data [χ ^2^ (143) = 4,868.60, *p* < .001, CFI = .96, RMSEA = .041 (CI: 0.040–0.042), SRMR = .03]. The model accounted for 60% of the variance in cognitive function at T3.

**Table 3.** Structural equation modeling Mediation Results for Cognition Function at T3 as an Outcome, Social Activities as the T1 Independent Variable and Physical Activity and Mental Health as Mediators at T2

Regression path	*B* (95% CI)	*B*
Pathways from social activities		
Social activities T1 → Physical activity T2	0.37 (0.16, 0.57)	0.07***
Social activities T1 → Mental health T2	2.73 (1.78, 3.69)	0.08***
Social activities T1 → Cognitive function T3	0.37 (0.11, 0.64)	0.04**
Pathways to cognitive function		
Cognitive function T1 → Cognitive function T3	0.61 (0.59, 0.64)	0.57***
Physical activity T2 → Cognitive function T3	0.06 (0.01, 0.10)	0.03*
Mental health T2 → Cognitive function T3	0.02 (0.01, 0.03)	0.07***

* *p* < .05, ** *p* < .01, *** *p* < .001.

The structural model showed that baseline social activities positively and significantly predicted cognitive functioning 4 years later, at T3, after controlling for sociodemographic and health covariates. The results indicated a total significant direct effect of social activities on cognitive functioning, after taking into account both physical activities and mental health. That is, being socially active at baseline was related to better future cognitive function, even while taking into account the mediating effects.

The results also indicated the existence of indirect effects. Social activities at baseline were positively related to better mental health and more physical activity at T2. Both mental health and physical activity at T2 were related to better cognitive function at T3. Analyses of indirect effects showed that social activities improved cognition by increasing physical activity frequency [*B* (*SE*) = 0.02 (0.01), *p* = .045]. Social activities also led to better cognitive functioning via improved mental health [*B* (*SE*) = 0.06 (0.01), *p* < .001]. In summary, increases in both physical activity and improvements in mental health mediated the effects of social activities on better cognition.

The analysis used the three cognitive variables as a latent factor. However, it is possible that these variables loaded on a single factor because they are all measures with verbal elements. Thus, additional analyses were run (not shown) to examine each cognitive component as a separate outcome in three separate models. All three models presented a similar trend of a direct effect of social activities on cognitive function and mediation through mental health and physical activities. This indicates the relevance of the current results for domains of memory as well as executive function.

## Discussion and Implications

This study examined the relationship between social activities and cognitive functioning and their mediating pathways. The results demonstrated that adults who engaged in social activities also had better cognitive health 4 years later. Additionally, the study found that social activities can impact cognition indirectly. Persons who were socially active engaged in more physical activity over time, which was related to better subsequent cognitive function. Socially active persons also had better mental health, which was related to improved cognition over time. Thus, these findings suggest that social activities can act on cognitive health through a physical avenue by improving physical activity and through a psychological avenue by improving mental health. Showing such mediation makes an important contribution to the understanding of the means through which the social environment affects cognition.

Engagement in social activities was shown to be directly associated with better cognitive performance over time, corroborating the study’s first hypotheses. In accordance with the “use it or lose it” theory, frequently engaging in social activities may operate as a form of mental exercise and help maintain cognitive abilities. The complexity of the social environment might contribute to cognitive reserve and brain reserve, which allows some people to cope with brain pathology better than others. Social activities can activate and strengthen different neurobiological pathways and functions or induce a more efficient use of brain networks (Scarmeas & Stern, 2003). The logistics of coordination (e.g., timing, transport), for instance, can contribute to cognitive reserve by providing opportunities for cognitive stimulation (Haslam et al., 2014). Group engagement typically implicates interacting with multiple people, many of whom are friends or not previously known, possibly providing additional sources of stimulation and new topics of conversation (Schwartz & Litwin, 2019a).

Additionally, the current study found that social activities can impact cognition indirectly, in accordance with the cascading causal process model of social integration and health (Berkman et al., 2000) and the model offered by Flatt and Hughes (2013). Thus, these findings shed light on the benefits of a socially active lifestyle in later life. The first mechanism through which social activities were shown to be associated with cognitive health is via increased physical activity, in support of the second study hypothesis. This finding underscores the notion that social activities can encourage physical exercise. Such activities can entail going out of the house and moving, and they can provide companions with whom one can do sport. Moreover, such social involvement can promote an overall active lifestyle, ultimately leading to better cognitive performance (Chen, Putnam, Lee, & Morrow-Howell, 2018).

Social activities were also shown to be associated with cognitive health through a psychological avenue, by improving mental health, corroborating the study’s third hypothesis. Frequently engaging in social activities may provide various emotional benefits, such as a sense of belonging and meaning, and the social group members can aid in coping with stressful events (Jang, Mortimer, Haley, & Graves, 2004; Prieto-Flores, Fernandez-Mayoralas, Forjaz, Rojo-Perez, & Martinez-Martin, 2011; Schwartz & Litwin, 2019b; Thoits, 2011). Mental health has important implications for cognition (Hertzog et al., 2009). These effects might be particularly pronounced in volunteer organizations and social groups that provide support to their members, as such support can promote volunteer commitment and maximize the social and mental health benefits experienced by older participants (Tang, Choi, & Morrow-Howell, 2010). Previous research showed that activity-centered interventions improve mental health by encouraging more social engagement (Juang et al., 2018). Arguably, such interventions might also have a positive impact on their participants’ cognitive health.

These findings can guide practitioners and policymakers in developing and optimizing social interventions aimed at older adults’ cognitive health. Therefore, when developing such socially oriented interventions, an emphasis should be placed on their physical and emotional aspects. These activities should be socially engaging, physically stimulating, enjoyable, and create an enriching environment, in accordance with the enrichment hypothesis (Hertzog et al., 2009). Activities such as hiking groups, team sports, or volunteering in sports organizations could be particularly fruitful, as adults engaging in them can enjoy both physical exercise and companionship (Pedersen et al., 2017; Steadman, Nykiforuk, & Vallianatos, 2013). Moreover, even social activities that are not explicitly focused on physical activity could be encouraged to incorporate such a component. For example, senior centers can include activities such as walking trips and working in the garden to keep older participants physically active. It is noteworthy that the intensity of exercise should be modified to appropriately match the older individual’s physical capability (McPhee et al., 2016). Frail older adults can benefit from moderate-intensity exercises, such as walking, and game-like activities that could be incorporated into social activities (Weening-Dijksterhuis, de Greef, Scherder, Slaets, & van der Schans, 2011).

The organizers of social activities should also be encouraged to address their participants’ psychological health. They can emphasize the social bonds created in these organizations and to promote emotionally meaningful activities. For example, participants in a knitting club can be encouraged to work towards donating their works to a meaningful cause which will make them feel valued and appreciated.

Health professionals may also use these findings to encourage older adults to become more socially active. As people grow older, they tend to worry and fear cognitive decline (Barber, 2017). Therefore, they might be particularly open to the notion that lifestyle changes can help delay age-related cognitive decline (Hertzog et al., 2009). They should be encouraged to participate in social activities as a means of maintaining a cognitively enriching lifestyle that can help them better maintain their cognitive health. The pathways linking social activities and cognition should be emphasized, such that older adults can work toward gaining physical and psychological benefits from their activities. Such messages might be particularly relevant for older individuals, as they tend to view physical activity as a means to socialize and develop relationships with meaningful others (Steltenpohl, Shuster, Peist, Pham, & Mikels, 2018). The current study focused on exploring the mediating mechanisms; however, a future study could examine the circumstances under which certain pathways are more relevant.

Some limitations of this study should be noted. One limitation is the issue of reverse causation, as it might be possible that better cognitive health leads to more social activities. However, several previous studies have found little evidence of reverse causation in the social activities–cognition relationship (Hertzog et al., 2009; James et al., 2011). Moreover, the longitudinal nature of the analyses strengthens the claim that being socially active can impact future cognitive functioning. Additionally, the current study is limited to general measures of social activities participation. The available data did not include information on the specific physical and mental aspects of the social activities. A more detailed elaboration of the underlying mechanisms could be obtained in the future using data such as the amount and type of physical exercise included in the social activity and the specific psychological benefits gained from it. It is also noteworthy that the current study measured the three cognitive variables as a single latent factor, and it is possible that they loaded on a single factor partly because of their verbal component. Even though additional analyses showed the relevance of the results for the cognitive variables when measured separately, it should be mentioned that other cognitive tasks with a verbal component might have similarly loaded on the latent cognitive variable. Another limitation is that most of the sample had a high level of education (at least secondary). Thus, although this was accounted for in the modeling, the results might differ among less-educated samples.

Furthermore, while the current study highlighted the protective effect of social activities, it did not address the modifiability of these activities and measured only initial level of activities as predictive of changes in cognition. As previous research has indicated that social activities can be modified (e.g., Tan et al., 2006), future studies should specifically examine changes in social activities (e.g., by studying social activity interventions) and their pathways to cognitive function. Also, additional pathways could exist between social activities and cognition, other than the factors explored in the current study. It might be possible that part of the direct effect of social activities reflects a process not currently explored. Future research can examine other mechanisms that underlie the connection between social activities and cognition, which were not available in the current data set, such as self-esteem or affect (Flatt & Hughes, 2013). Another possibility can be to examine the vascular hypothesis, which claims that social activities can act via beneficial effects on cardiovascular diseases and stroke (Fratiglioni, Paillard-Borg, & Winblad, 2004). It is also possible that the mediational mechanisms differ among age groups, and a future investigation may add valuable insight to the current findings by examining whether these pathways are found equally for young-old and old-old adults.

In conclusion, this study provides evidence regarding the mechanisms underlying the effects of social activities on older individuals’ cognitive health. It suggests that social activities are related to improved cognition via better mental health and physical activities. However, even after taking these pathways into account, social activities had a direct impact on cognitive functioning, possibly suggesting that the social contacts themselves entail cognitive stimulation that facilitates cognitive health. These results point toward policy and practice implications of promoting social activities among the older population as a meaningful avenue of improving cognitive aging. The results, furthermore, underscore the emotional and physical mechanisms underlying these effects, emphasizing the need to pay attention to these aspects of social activities in the older population. The beneficial effects of such activities should be brought to the public’s attention as facilitating a more positive aging process.
